# The Association between ADIPOQ Gene Polymorphisms and Diabetic Retinopathy Risk: A Systematic Review and Meta-Analysis

**DOI:** 10.3390/medicina60081254

**Published:** 2024-08-01

**Authors:** Konstantinos Flindris, Georgios Markozannes, Marilita Moschos, Maria Gazouli, Aikaterini Christodoulou, Konstantinos Tsilidis, Georgios Kitsos

**Affiliations:** 1 Department of Ophthalmology, University General Hospital of Ioannina, 45500 Ioannina, Greece; xristodoulouk@yahoo.gr (A.C.); gkitsos@uoi.gr (G.K.); 2 Department of Hygiene and Epidemiology, Medical School, University of Ioannina, 45500 Ioannina, Greece; georgemarkozannes@gmail.com (G.M.); ktsilidis@gmail.com (K.T.); 3 1st Department of Ophthalmology, University of Athens, 11527 Athens, Greece; moschosmarilita@yahoo.fr; 4 Laboratory of Biology, Department of Basic Medical Sciences, Medical School, National and Kapodistrian University of Athens, 11527 Athens, Greece; maria.gazouli@gmail.com

**Keywords:** diabetic retinopathy, adiponectin gene, ADIPOQ gene, rs2241766, rs1501299

## Abstract

*Background and Objectives*: Recent studies have focused on the association between the risk of diabetic retinopathy (DR) and the rs1501299 and rs2241766 polymorphisms of the ADIPOQ gene; however, their results remain inconclusive. Thus, a systematic review and meta-analysis were carried out to clarify the role of these polymorphisms in the development of DR. *Materials and Methods*: A systematic search of electronic databases (PubMed, Scopus, and Cochrane Library) was conducted until 25 June 2024, and a reference list of relevant articles was collected, which explored the association between the rs1501299 and rs2241766 polymorphisms of the ADIPOQ gene and the risk of DR. The pooled odds ratios (OR) and 95% confidence intervals (CI) were estimated via random-effects model, and the meta-analysis was implemented by using Review Manager 5.4. *Results*: In total, 6 out of 182 studies, with 1888 cases (DR) and 2285 controls (without DR), were included in the meta-analysis. A statistically significant association between the rs1501299 polymorphism and the DR risk was recorded in G vs. T in the overall analysis (OR = 0.84, 95% CI = 0.72–0.99, *p* = <0.05, I^2^ = 23%, *n* = 5 studies). Additionally, the summary results in the subgroup analysis according to the control type were as follows: the DR versus diabetes mellitus (DM) control type revealed a statistically significant association in G vs. T: OR = 0.81, 95% CI = 0.67–0.97, *p* = <0.05, I^2^ = 27%, *n* = 4 studies; GG vs. GT: OR = 0.72, 95% CI = 0.53–0.98, *p* = <0.05, I^2^ = 49%, n = 4 studies; GG vs. (GT + TT): OR = 0.73, 95% CI = 0.55–0.96, *p* = <0.05, I^2^ = 44%, *n* = 4 studies. No significant association was observed between the rs2241766 polymorphism and the DR risk. *Conclusions*: The current meta-analysis supports the association between the rs1501299 polymorphism of the ADIPOQ gene and the DR risk in patients with DM.

## 1. Introduction

Diabetic retinopathy (DR) is a common microvascular and neurodegenerative complication of diabetes mellitus (DM) and remains the leading cause of preventable vision loss in working-aged people (20–74 years) in developed countries [1]. To date, three out of four people with DM have signs of DR after 15 years of disease duration, reaching 93 million worldwide, while an increase in these cases by 150% is expected by 2040 [2]. According to the severity of the disease, DR is classified into non-proliferative diabetic retinopathy (NPDR) and proliferative diabetic retinopathy (PDR). The signs of NPDR include microaneurysms, cotton wool spots, hard exudates, intraretinal vascular abnormalities, and venous beading, and this condition can progressively develop into PDR, which is characterized by neovascularization and preretinal or vitreous hemorrhage [3]. As a matter of fact, DR is harmful for individuals and society due to the imposed health, psychological, and financial burdens [4].

The etiopathogenesis of DR has been studied thoroughly [5,6], but the precise underlying mechanisms have not been clarified, and the currently available therapies are insufficient to prevent or minimize the ocular complications of DM [7]. The main risk factors are DM duration and poor glycemic control, while several other risk factors have been identified, including the presence of arterial hypertension, dyslipidemia, obesity, cardiovascular disease, stroke, nephropathy, smoking, pregnancy, anemia, and cataract surgery [8]. Chronic hyperglycemia leads to intracellular oxidative stress through sundry overarching biochemical pathways, inducing vascular hyperpermeability through endothelium cells and pericyte deaths and promoting neuroglia dysfunction, degeneration of retinal neurons, and inflammation and leukostasis in the diabetic retina [9]. These pathogenic pathways result in hypoperfusion, local ischemia, and neovascularization, which ultimately give rise to PDR and diabetic macular edema (DME) [10].

In addition, the prevalence of DR varies significantly between different ethnicity groups and is higher in people with type 1 DM (T1DM) rather than type 2 DM (T2DM), due to the longer duration of the disease [11]. However, there is an important variation in DR risk, and the complexity of the disease may be explained by genetic factors, such as gene mutations and abnormal expression, which play a significant role in the onset and progression of DR [12].

Adiponectin, a product of the ADIPOQ human gene, is expressed in adipose tissue and modulates sundry metabolic processes, including glucose regulation and lipid metabolism [13]. This adipokine exerts anti-inflammatory and anti-atherogenic effects, as it can prevent vascular remodeling by inhibiting the proliferation and migration of smooth muscle cells, and it can reduce the Tumor Necrosis Factor-a (TNF-a) production to modulate the inflammatory response of endothelial cells In fact, adiponectin can protect the vasculature through its pleiotropic actions on endothelial progenitor cells, endothelial cells, macrophages, and smooth muscle cells, and it may prevent the injury and dysfunction in endothelial cells. The ADIPOQ gene is located on chromosome 3q27 and consists of three exons and two introns, spanning a 17 kb region [14]. Genetic polymorphisms of this gene may affect the plasma adiponectin levels and their contribution to the occurrence of DR [15]. Two common single nucleotide polymorphisms (SNPs) in the ADIPOQ gene locus at 276G/T (rs1501299) and 45T/G (rs2241766) have been studied in different ethnic groups and have been found to be associated with T2DM and its complications [16,17,18,19]. 

Many studies have been carried out to investigate the association between these two SNPs and the risk of DR, but their results were conflicting and inconclusive [20,21,22,23,24,25]. Thus, the aim of this systematic review and meta-analysis is to investigate the association between ADIPOQ polymorphisms rs1501299 and rs2241766 and the development of DR.

## 2. Materials and Methods

This study was conducted according to a predetermined protocol, which was registered in PROSPERO on 21/09/2022 (ID: CRD42022361770). This systematic review and meta-analysis were performed based on the Preferred Reporting Items for Systematic Reviews and Meta-analyses (PRISMA) guidelines [26]. Study identification and selection, data extraction, and quality assessment were carried out independently by two researchers (K. F. and G. M.), and disagreement was settled through discussion. If no consensus was reached, a third researcher made the ultimate decision (G. K.).

### 2.1. Search Strategy

The online databases of PubMed, Scopus, and Cochrane Library were searched up to 25 June 2024, for all potentially relevant publications in English on the association between the ADIPOQ rs2241766 and rs1501299 gene polymorphisms and DR risk. The following algorithm was used for searching: *“(Adiponectin OR ADIPOQ) AND (rs2241766 OR rs1501299 OR polymorphism OR single nucleotide polymorphism OR SNP OR variant OR variation OR mutation) AND (Diabetic microangiopathy OR Diabetic retinopathy OR DR)”*. The reference list of the retrieved articles was hand-searched.

### 2.2. Eligibility Criteria

The included studies consisted of case–control and cohort studies on the association between ADIPOQ rs1501299 and rs2241766 gene polymorphisms and DR development and had sufficient published data to estimate an odds ratio (OR) and 95% confidence intervals (95% CI) for each allele or genotype. The genotype distribution of these polymorphisms was determined in cases of diabetic retinopathy of any grade (NPDR or PDR) in T1DM or T2DM and in controls. The control group consisted of healthy controls (HC) or individuals with DM but free of DR (NDR). The study subjects were exclusively humans. A minimum number of participants for a study to be included in the meta-analysis was not defined. Furthermore, all participants of the included studies provided informed consent for their participation in the original studies, and these studies were approved by the ethics committees of the participating institutions.

The exclusion criteria were: (1) reviews, editorials, abstracts, case reports, and animal studies; (2) studies with inadequate or overlapping data on allele and genotype frequencies; (3) studies in which the genotype distribution of the controls deviated from the Hardy–Weinberg equilibrium (HWE).

The HWE of the genotype distribution of the controls was tested through a χ^2^ analysis, and a *p*-value > 0.05 suggested that the study sample was representative of the population [27].

### 2.3. Data Extraction

After the selection process, data were extracted regarding the following characteristics of the included articles: name of the first author, year of publication, country, ethnicity of the population, study design, genotyping method, DM type, control type, demographic/clinical characteristics, and sample sizes of cases and controls for the ADIPOQ rs1501299 and rs2241766 genotypes. The allele frequencies and the genotypic distribution were calculated or extracted for both the case and control group. Moreover, the minor allele frequency (MAF) for ADIPOQ rs1501299 and rs2241766 polymorphisms was calculated, and we examined whether the distribution of genotypes in the control group in each study was consistent with the HWE [28]. 

### 2.4. Quality Assessment

The Newcastle–Ottawa Scale (NOS) was used to evaluate the quality of the included observational studies. The NOS consists of 3 parts: selection (4 entries), comparability (1 entry), and exposure (3 entries) for the case–control studies and selection (4 entries), comparability (1 entry), and outcome (3 entries) for the cohort studies. Each entry received only one star for a quality item in the selection and exposure/outcome parts, and a maximum of two stars was awarded in the comparability part for each study. The NOS is a semi-quantitative scale, and the score ranges from 0 (worst) to 9 (best) stars for each included study. Studies with 7 or more stars are considered to be of relatively high quality [29]. 

### 2.5. Statistical Analysis

The meta-analysis was conducted using Review Manager 5.4 (The Cochrane Collaboration, Oxford, UK). The following genotype contrasts were assessed for the ADIPOQ rs1501299 polymorphism: homozygotes GG versus a combination of GT and TT [GG vs. (GT + TT), dominant model], a combination of GG and GT versus TT [(GG + GT) vs. TT, recessive model], GG versus TT, GT versus TT, GG versus GT, and a combination of GG and TT versus GT [(GG + TT) vs. GT, over-dominant model]. The G allelic frequency versus the T allelic frequency was also examined (G versus T). In addition, for the ADIPOQ rs2241766 polymorphism the following genotype contrasts were evaluated: TT versus a combination of TG and GG [TT vs. (TG + GG), dominant model], a combination of TT and TG versus GG [(TT + TG) vs. GG, recessive model], TT versus GG, TG versus GG, TT versus TG, a combination of TT and GG versus TG [(TT + GG) vs. GT, over-dominant model], and T allele versus G allele [30].

The summary odds ratio and 95% confidence interval were estimated via the random-effects DerSimonian–Laird model [31]. Heterogeneity was assessed through the χ^2^-based Q statistic test, and it was considered statistically significant for *p*-value < 0.05. The degree of heterogeneity was quantified by the I^2^ metric, which ranges from 0% to 100%, with higher values indicating a greater degree of between-study variability that can be attributed to heterogeneity. Furthermore, subgroup analyses by ethnicity and control type were performed. Additionally, meta-regression analyses were performed to identify the sources of heterogeneity if at least 10 studies were available. Sensitivity analysis was used to examine the stability of the results by gradually removing the included studies one by one. The potential publication bias was assessed using the Begg adjusted rank correlation test and the Egger regression asymmetry test if at least 10 studies were available. Statistical significance was set at *p*-value < 0.05, and all *p*-values were two-sided [32]. 

## 3. Results

### 3.1. Literature Search

**Figure 1:**
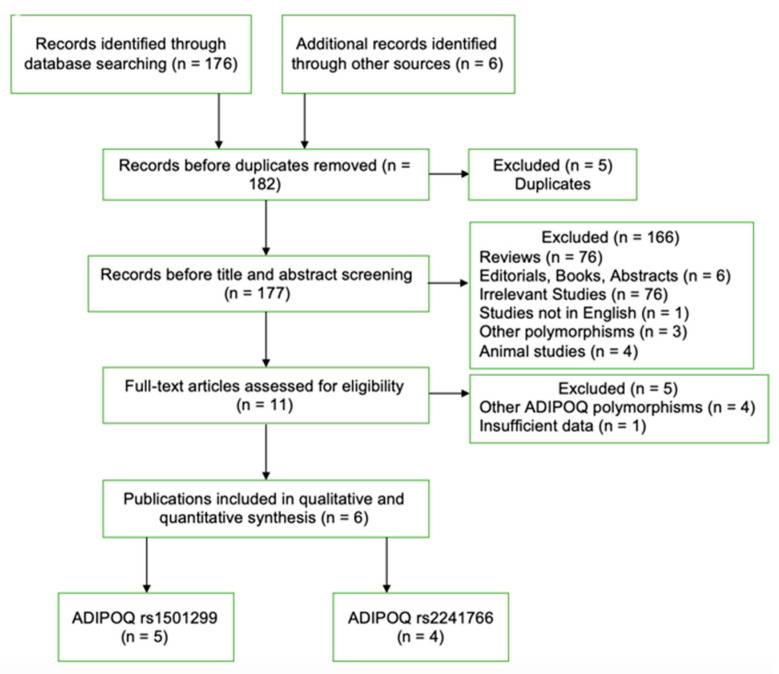
Flow diagram of the study selection process. From the initial search, 182 citations were identified; ultimately, a total of six publications were included in the meta-analysis, five of which analyzed the rs1501299 polymorphism, and four of which investigated the rs2241766 polymorphism.

The process of the study selection is presented in Figure 1. In the initial search, a total of 182 citations were identified. After the exclusion of five duplicates, another 177 citations were excluded based on title and abstract screening, since they did not meet the inclusion criteria. Then 11 full-text articles were assessed for eligibility, and five citations were excluded [33,34,35,36,37]. Finally, a total of six publications were included in the systematic review involving a total of 1888 patients and 2285 controls, five of which investigated rs1501299 polymorphism with 1014 cases and 1234 controls [20,21,23,24,25] and four of which explored rs2241766 polymorphism with 874 and 1051, respectively [21,22,23,24]. 

### 3.2. Study Characteristics and Summary Statistics

The eligible study characteristics and the genotype and allelic distribution of ADIPOQ rs1501299 and rs2241766 SNPs are shown in Table 1 and Table 2. All the studies were based on T2DM, and in four of them the population was Asian [22,23,24,25], while in the other two, the population was Caucasian [20,21]. All the studies had a case–control study design [20,21,22,24,25], except for one that had a cohort study design [23]. In one study, the control group consisted of healthy participants [20], three studies consisted of participants with T2DM but without signs of DR (NDR) [21,23,24], and, finally, in two studies, there were both healthy participants and NDR [22,25]; thus, two different associations were investigated.

The NOS scores of the included studies ranged from 6 to 8, with a median of 7 (Table 2). Five studies were considered of high quality [20,21,23,24,25], while the other was of mediocre quality [22]. All studies indicated that the distribution of genotypes in the control group was consistent with the HWE (Table 2).

### 3.3. Quantitative and Subgroup Analyses

#### 3.3.1. Association between rs1501299 Polymorphism and DR Risk

**Figure 2:**
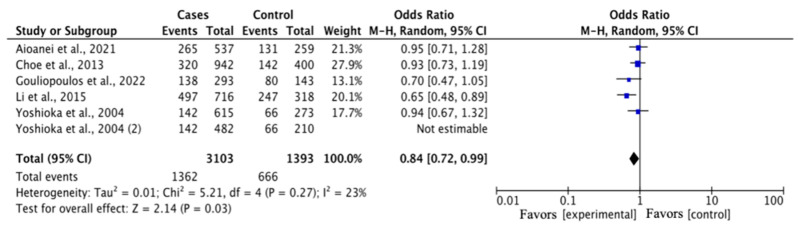
Forest plot of the rs1501299 polymorphism and DR risk in the overall analysis in the allelic contrast, illustrating a statistically significant association in favor of the cases [20,21,23,24,25].

**Figure 3:**
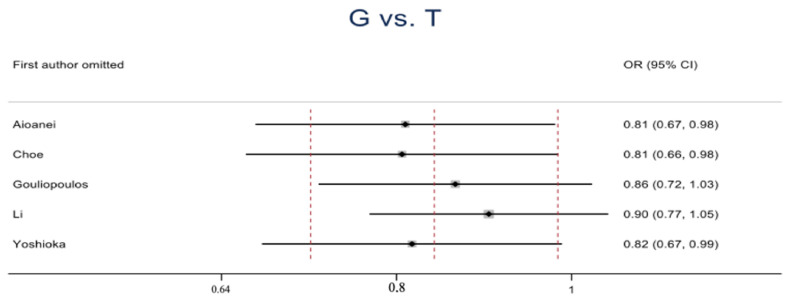
Sensitivity analysis through deletion of one study at a time for the rs1501299 polymorphism and DR risk association in the overall comparison in the allelic contrast presenting a minor change when excluding the studies.

The estimated results of the association between the rs1501299 polymorphism and DR risk are shown in Table 3. In the overall analysis, a statistically significant association was observed in G vs. T (OR = 0.84, 95% CI = 0.72–0.99, *p* < 0.05) (Figure 2), a sensitivity analysis through deletion of one study at a time was conducted to reflect the influence of the individual dataset to the pooled OR, and a minor change was observed when excluding the studies (Figure 3). The other genetic comparisons reported no significant difference.

Subgroup analysis was performed according to ethnicity and control type. The results of the analysis performed on Asian populations showed no statistically significant association in any genetic model, as well as on Caucasian populations (Table 3).

**Figure 4:**
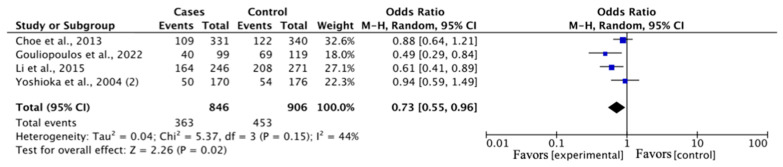
Forest plot of the rs1501299 polymorphism and DR risk in the subgroup comparison based on DR versus DM control type in the dominant genetic model, demonstrating a statistically significant association in favor of the cases [21,23,24,25].

The pooled results in the DR versus DM control type revealed a statistically significant association in G vs. T: OR = 0.81, 95% CI = 0.67–0.97, *p* = <0.05; GG vs. GT: OR = 0.72, 95% CI = 0.53–0.98, *p* = <0.05; GG vs. (GT + TT): OR = 0.73, 95% CI = 0.55–0.96, *p* = <0.05 (Figure 4). The other genetic models in this analysis presented no significant association. The pooled results in the DR versus HC showed no significant association in any genetic model (Table 3).

#### 3.3.2. Association between the rs2241766 Polymorphism and DR Risk

Furthermore, the pooled results indicated a non-association between rs2241766 ADIPOQ gene polymorphism and susceptibility to DR in the overall or subgroup analyses (ethnicity, control type) in any genetic model (Table 4). However, the Caucasian population subgroup and the DR versus HC subgroup consisted of one study each; hence, meta-analysis was not feasible.

## 4. Discussion

The present meta-analysis of five studies for the rs1501299 polymorphism, including 1014 cases and 1234 controls, provided an elucidative analysis of the association of this SNP with DR risk. The results indicated a statistically significant association between the rs1501299 polymorphism and the DR risk in the overall studied population in the allelic contrast. This estimate is consistent with all the included studies in the meta-analysis [20,21,23,24,25]. In the subgroup analysis according to ethnicity, no significant association was recorded in the Asian and Caucasian populations in all genetic models, due to the small sample, especially in the second case. In the subgroup analysis according to the control type, statistically significant associations were observed when the control group consisted of patients with DM but without signs of DR, in the allelic contrast, in the dominant genetic model and in GG vs. GT. This may occur because a comparison between a group with DR and a group with DM but without DR may reduce the confounding factor of the common background disease and possibly uncover any responsible genetic predisposition [21,23,24,25].

Although the results of this meta-analysis support the association between the rs1501299 polymorphism of the ADIPOQ gene and DR risk in patients with DM, the results presented are potentially burdened by biases, since the included case–control studies did not adequately adjust for the prognostic and confounding factors, which may influence the outcome independently from the SNP studied. In fact, one study matched the participants by age and gender [21], and another study matched them by ethnicity and gender [22]. As a consequence, extensive and rigorous original studies are needed in order to elucidate the precise association between the rs1501299 polymorphism and DR risk.

The meta-analysis of four studies for the rs2241766 polymorphism of the ADIPOQ gene, including 874 cases and 1051 controls, found no statistically significant association with the risk of DR, in the overall or the subgroup analyses, in any genetic model. This may be partly explained due to the small number of included studies and the relatively small sample sizes. The leave-one-out sensitivity analyses showed that the results were stable and unaffected by single studies.

DR is the most serious ocular complication of DM and is the most common vascular disease of the retina, which can lead to vision loss if left untreated [38,39]. The prevalence of DR varies depending on several factors, such as genetic background and the duration of diabetes and the level of glycemic control [40]. The global prevalence of diabetic retinopathy among people with diabetes is estimated to be around 27%, with the highest prevalence rates reported in low- and middle-income countries due to the late diagnoses of DM leading to untreated disease [41].

Previous studies documented that the rs1501299 GT genotype could be recognized as an independent risk factor of DR, while no association between rs2241766 and DR was identified in a Caucasian population [21]. On the other hand, a higher risk of DR was indicated in the rs2241777 TT genotype in an Asian population [22]. Hence, a systematic review and meta-analysis was carried out to elucidate the association between ADIPOQ polymorphisms rs1501299 in intron 2 and rs2241766 in exon 2 and the DR risk.

In previous studies, the rs1501299 polymorphism of the ADIPOQ gene has been correlated with T2DM, cardiovascular diseases, metabolic syndrome, hepatocellular carcinoma, and endometrial cancer [42,43,44,45,46]. The rs2241766 genetic polymorphism has been associated with T2DM, hypercholesterolemia, obesity, metabolic syndrome, and cardiovascular diseases [43,47,48,49,50,51]. In fact, these two SNPs may be associated with the background disease and with the risk factors of DR, contributing indirectly to the onset of DR. Therefore, their targeted intervention may reduce the burden of the disease for the patient and for the health system, through effective and efficient gene therapy.

This study had some limitations that should be taken into consideration when interpreting the results. Firstly, only published studies in English were included in the quantitative analysis, while potential studies in other languages could have been omitted. However, most studies were in Asian populations; so, the potential for missing studies is reduced. Secondly, the meta-analysis was based predominantly on Asian studies, while only two studies of the Caucasian population were included, no study from another part of the world was identified, and the number of study participants was limited. Consequently, this may hinder the generalizability of the results; however, the sub-group analysis based on the ethnicity of the participants was conducted, and the OR did not differ significantly. Thirdly, confounding exogenous factors such as age, sex, and lifestyle factors were not adjusted in all studies, since one study matched the participants by age and gender [21] and another study matched them by ethnicity and gender [22], although their effect on the SNPs’ expression may be of minor importance. Last but not least, the OR and 95% CI of the included studies were estimated based on the genotype distribution, since these data were not present in all of the studies. Despite these limitations, this systematic review and meta-analysis provides a better understanding of the association between the rs1501299 and rs2241766 polymorphisms of the ADIPOQ gene and the risk of DR in T2DM.

## 5. Conclusions

To summarize, the current meta-analysis provides evidence supporting the association of the rs1501299 polymorphism and DR risk in patients with T2DM, while the rs2241766 polymorphism showed no association with the development of DR. Considering the limitations of this study, further larger and more rigorous studies with different ethnic populations are required to verify these findings and clarify the relationship between DR and genetic heritage.

## Figures and Tables

**Table 1:** Characteristics of the included studies on ADIPOQ gene polymorphisms and diabetic retinopathy risk.

First Author	Year of Publication	Country/Ethnicity	Study Design	Genotyping Method	Control Type	Type of DM	DR Grade	Case		
								Sample Size	Sex (M/F)	Age (years)
Aioanei	2021	Romania/Caucasian	Case–Control	PCR-RFLP	HC	T2DM	NPDR	198	105/93	68.72 ± 11.58
Choe	2013	Korea/Asian	Cohort Study	PCR-RFLP	NDR	T2DM	Any DR	231	N/A	N/A
Gouliopoulos	2022	Greece/Caucasian	Case–Control	PCR-RFLP	NDR	T2DM	Any DR	109	74/35	67.00 ± 8.00
Li	2015	China/Asian	Case–Control	PCR-RFLP	NDR	T2DM	Any DR	372	146/226	63.39 ± 10.60
Yoshioka	2004	Japan/Asian	Case–Control	PCR-RFLP	HC + NDR	T2DM	Any DR	104	55/49	62.05 ± 9.20
Choe	2013	Korea/Asian	Cohort Study	PCR-RFLP	NDR	T2DM	Any DR	225	N/A	N/A
Gouliopoulos	2022	Greece/Caucasian	Case–Control	PCR-RFLP	NDR	T2DM	Any DR	109	74/35	67.00 ± 8.00
Li	2015	China/Asian	Case–Control	PCR-RFLP	NDR	T2DM	Any DR	372	146/226	63.39 ± 10.60
Sikka	2014	India/Asian	Case–Control	PCR-RFLP	HC+NDR	T2DM	Any DR	169	N/A	58.35 ± 9.01

Abbreviations: DM, Diabetes mellitus; DR, Diabetic retinopathy; T2DM, Type 2 diabetes mellitus; NPDR, non-proliferative diabetic retinopathy; HC, healthy controls; NDR, non-diabetic retinopathy diabetes mellitus, PCR-RFLP, polymerase chain reaction—restriction fragment length polymorphism; M/F, male/female, N/A, not available.

**Table 2:** Characteristics of the included studies on ADIPOQ gene polymorphisms and diabetic retinopathy risk.

First Author	Control			Genotype Distribution						HWE *p*-Value	MAF		NOS (Stars)
	Sample Size	Sex (M/F)	Age (years)	Case			Control			Control	Case	Control	
				rs1501299 G/T									
				GG	GT	TT	GG	GT	TT				
Aioanei	200	143/57	58.10 ± 9.00	93	79	26	92	88	20	0.876	0.33	0.32	7
Choe	440	N/A	N/A	109	102	20	222	178	40	0.616	0.31	0.29	7
Gouliopoulos	109	75/34	66.00 ± 9.00	40	58	11	59	37	13	0.069	0.37	0.29	7
Li	145	49/96	62.34 ± 10.75	164	169	39	82	55	8	0.756	0.33	0.24	8
Yoshioka	340	219/121	59.70 ± 10.10	50	42	12	163	147	30	0.699	0.32	0.30	7
				rs2241766 T/G									
				TT	TG	GG	TT	TG	GG				
Choe	442	N/A	N/A	111	96	18	213	194	35	0.315	0.29	0.30	7
Gouliopoulos	109	75/34	66.00 ± 9.00	84	23	2	74	32	3	0.836	0.12	0.17	7
Li	145	49/96	62.34 ± 10.75	206	140	25	82	53	10	0.720	0.26	0.25	8
Sikka	355	N/A	53.16 ±12.15	158	9	2	292	58	5	0.285	0.04	0.09	6

Abbreviations: M/F, male/female; HWE, Hardy–Weinberg equilibrium, MAF, minor allele frequency, NOS, Newcastle–Ottawa scale; N/A, not available.

**Table 3:** Meta-analysis of the association of the ADIPOQ rs1501299 polymorphism and diabetic retinopathy risk.

rs1501299	Study	Sample Size		Studies (n)	Test of Association			Test of Heterogeneity			
		Cases	Controls		OR (95% CI)	Z	*p*-Value	χ^2^	*p*-Value	I^2^ (%)	T^2^
G vs. T	Overall	3103	1393	5	0.84 (0.72–0.99)	2.14	0.03	5.21	0.27	23	0.01
	Asian	2273	991	3	0.84 (0.66–1.05)	1.51	0.13	3.76	0.15	47	0.02
	Caucasian	830	402	2	0.84 (0.63–1.13)	1.14	0.25	1.44	0.23	31	0.01
	DR vs. DM	2433	1071	4	0.81 (0.67–0.97)	2.28	0.02	4.13	0.25	27	0.01
	DR vs. HC	812	388	2	0.97 (0.76–1.24)	0.21	0.83	0.07	0.79	0	0.00
GG vs. TT	Overall	1074	219	5	0.76 (0.55–1.04)	1.73	0.08	3.02	0.55	0	0.00
	Asian	790	149	3	0.72 (0.44–1.18)	1.32	0.19	2.99	0.22	33	0.06
	Caucasian	284	70	2	0.79 (0.46–1.33)	0.90	0.37	0.00	0.96	0	0.00
	DR vs. DM	846	165	4	0.75 (0.52–1.08)	1.55	0.12	3.01	0.39	0	0.00
	DR vs. HC	278	66	2	0.78 (0.45–1.34)	0.91	0.36	0.00	1.00	0	0.00
GT vs. TT	Overall	955	219	5	0.90 (0.63–1.29)	0.56	0.57	4.82	0.31	17	0.03
	Asian	693	149	3	0.87 (0.58–1.30)	0.70	0.48	1.70	0.43	0	0.00
	Caucasian	262	70	2	1.08 (0.41–2.81)	0.15	0.88	3.00	0.08	67	0.32
	DR vs. DM	741	165	4	1.00 (0.66–1.52)	0.01	0.99	3.64	0.30	18	0.03
	DR vs. HC	256	66	2	0.66 (0.38–1.14)	1.49	0.14	0.06	0.81	0	0.00
GG vs. GT	Overall	1074	955	5	0.80 (0.60–1.08)	1.44	0.15	9.55	0.05	58	0.07
	Asian	790	693	3	0.83 (0.64–1.08)	1.40	0.16	2.60	0.27	23	0.01
	Caucasian	284	262	2	0.71 (0.28–1.82)	0.71	0.48	6.94	0.008	86	0.39
	DR vs. DM	846	741	4	0.72 (0.53–0.98)	2.06	0.04	5.85	0.12	49	0.05
	DR vs. HC	278	256	2	1.18 (0.84–1.66)	0.97	0.33	0.16	0.69	0	0.00
GG vs. GT + TT	Overall	1074	1174	5	0.79 (0.61–1.03)	1.76	0.08	8.15	0.09	51	0.04
	Asian	790	842	3	0.81 (0.61–1.07)	1.48	0.14	3.34	0.19	40	0.02
	Caucasian	284	332	2	0.73 (0.35–1.52)	0.83	0.40	4.81	0.03	79	0.22
	DR vs. DM	846	906	4	0.73 (0.55–0.96)	2.26	0.02	5.37	0.15	44	0.04
	DR vs. HC	278	322	2	1.09 (0.79–1.50)	0.50	0.61	0.14	0.71	0	0.00
GG + GT vs. TT	Overall	2031	219	5	0.82 (0.61–1.12)	1.26	0.21	3.31	0.51	0	0.00
	Asian	1485	149	3	0.78 (0.51–1.19)	1.14	0.26	2.38	0.30	16	0.02
	Caucasian	546	70	2	0.87 (0.53–1.44)	0.53	0.59	0.85	0.36	0	0.00
	DR vs. DM	1587	165	4	0.86 (0.60–1.23)	0.81	0.42	3.06	0.38	2	0.00
	DR vs. HC	534	66	2	0.72 (0.43–1.21)	1.25	0.21	0.02	0.90	0	0.00
GG + TT vs. GT	Overall	1293	955	5	0.85 (0.63–1.13)	1.13	0.26	9.80	0.04	59	0.06
	Asian	939	693	3	0.87 (0.70–1.08)	1.23	0.22	1.99	0.37	0	0.00
	Caucasian	354	262	2	0.75 (0.29–1.91)	0.61	0.54	7.78	0.005	87	0.40
	DR vs. DM	1011	741	4	0.76 (0.57–1.02)	1.82	0.13	5.72	0.13	48	0.04
	DR vs. HC	344	256	2	1.24 (0.90–1.72)	1.30	0.19	0.16	0.69	0	0.00

Abbreviations: DM, diabetes mellitus, DR, diabetic retinopathy; HC, healthy-based controls.

**Table 4:** Meta-analysis of the association of the ADIPOQ rs2241766 polymorphism and diabetic retinopathy risk.

rs2241766	Study	Sample Size		Studies (n)	Test of Association			Test of Heterogeneity			
		Cases	Controls		OR (95% CI)	Z	*p*-Value	χ^2^	*p*-Value	I^2^ (%)	T^2^
T vs. G	Overall	3045	805	4	1.30 (1.00–2.09)	1.42	0.15	10.03	0.02	70	0.09
	Asian	2674	740	3	1.27 (0.82–1.96)	1.06	0.29	8.93	0.01	78	0.11
	DR vs. DM	2681	763	4	1.21 (0.90–1.64)	1.27	0.20	6.76	0.08	56	0.05
TT vs. GG	Overall	1220	100	4	1.34 (0.87–2.07)	1.31	0.19	1.74	0.63	0	0.00
	Asian	1062	95	3	1.32 (0.84–2.07)	1.21	0.23	1.66	0.44	0	0.00
	DR vs. DM	1056	97	4	1.33 (0.86–2.07)	1.28	0.20	1.74	0.63	0	0.00
TG vs. GG	Overall	605	100	4	0.94 (0.60–1.49)	0.26	0.79	1.06	0.79	0	0.00
	Asian	550	95	3	0.93 (0.58–1.50)	0.29	0.77	1.03	0.60	0	0.00
	DR vs. DM	569	97	4	0.96 (0.60–1.52)	0.18	0.86	0.70	0.87	0	0.00
TT vs. TG + GG	Overall	1220	705	4	1.38 (0.89–2.15)	1.45	0.15	10.61	0.01	72	0.14
	Asian	1062	645	3	1.35 (0.78–2.35)	1.07	0.28	9.69	0.008	79	0.18
	DR vs. DM	1056	666	4	1.29 (0.89–1.87)	1.35	0.18	7.21	0.07	58	0.08
TT vs. TG	Overall	1220	605	4	1.41 (0.88–2.25)	1.43	0.15	10.92	0.01	73	0.16
	Asian	1062	550	3	1.39 (0.76–2.53)	1.08	0.28	10.13	0.006	80	0.22
	DR vs. DM	1056	569	4	1.31 (0.88–1.95)	1.33	0.18	7.54	0.06	60	0.09
TT + TG vs. GG	Overall	1845	100	4	0.99 (0.64–1.54)	0.03	0.97	0.33	0.95	0	0.00
	Asian	1632	95	3	0.97 (0.62–1.52)	0.14	0.88	0.11	0.95	0	0.00
	DR vs. DM	1645	97	4	0.99 (0.63–1.53)	0.06	0.95	0.30	0.96	0	0.00
TT + GG vs. TG	Overall	1320	605	4	1.40 (0.88–2.21)	1.42	0.16	10.91	0.01	72	0.15
	Asian	1157	550	3	1.38 (0.77–2.49)	1.08	0.28	10.14	0.006	80	0.21
	DR vs. DM	1153	569	4	1.30 (0.88–1.92)	1.31	0.19	7.51	0.06	60	0.09

Abbreviations: DM, diabetes mellitus, DR, diabetic retinopathy.

## Data Availability

Data available upon request.

## Abbreviations

DR	Diabetic retinopathy
OR	Odds ratio
CI	Confidence interval
DM	Diabetes mellitus
NPDR	Non-proliferative diabetic retinopathy
PDR	Proliferative diabetic retinopathy
DME	Diabetic macular edema
T1DM	Type 1 diabetes mellitus
T2DM	Type 2 diabetes mellitus
TNF-a	Tumor necrosis factor—a
SNP	Single nucleotide polymorphisms
PRISMA	Preferred Reporting Items for Systematic Reviews and Meta-analyses
HC	Healthy controls
NDR	Non-diabetic retinopathy
HWE	Hardy–Weinberg equilibrium
MAF	Minor allele frequency
NOS	Newcastle–Ottawa scale
PCR-RFLP	Polymerase chain reaction—restriction fragment length polymorphism
N/A	Not available
